# Functional connectivity changes during a working memory task in rat via NMF analysis

**DOI:** 10.3389/fnbeh.2015.00002

**Published:** 2015-01-30

**Authors:** Jing Wei, Wenwen Bai, Tiaotiao Liu, Xin Tian

**Affiliations:** ^1^School of Biomedical Engineering, Tianjin Medical UniversityTianjin, China; ^2^Research Center of Basic Medicine, Tianjin Medical UniversityTianjin, China

**Keywords:** working memory (WM), spikes, functional connectivity, dimensionality reduction, non-negative matrix factorization (NMF)

## Abstract

Working memory (WM) is necessary in higher cognition. The brain as a complex network is formed by interconnections among neurons. Connectivity results in neural dynamics to support cognition. The first aim is to investigate connectivity dynamics in medial prefrontal cortex (mPFC) networks during WM. As brain neural activity is sparse, the second aim is to find the intrinsic connectivity property in a feature space. Using multi-channel electrode recording techniques, spikes were simultaneously obtained from mPFC of rats that performed a Y-maze WM task. Continuous time series converted from spikes were embedded in a low-dimensional space by non-negative matrix factorization (NMF). mPFC network in original space was constructed by measuring connections among neurons. And the same network in NMF space was constructed by computing connectivity values between the extracted NMF components. Causal density (Cd) and global efficiency (E) were estimated to present the network property. The results showed that Cd and E significantly peaked in the interval right before the maze choice point in correct trials. However, the increase did not emerge in error trials. Additionally, Cd and E in two spaces displayed similar trends in correct trials. The difference was that the measures in NMF space were significantly greater than those in original space. Our findings indicated that the anticipatory changes in mPFC networks may have an effect on future WM behavioral choices. Moreover, the NMF analysis achieves a better characterization for a brain network.

## Introduction

Working memory (WM) has been defined as a limited capacity system which temporarily maintains and manipulates information “online” during various cognitive tasks (Baddeley, 2003). It supports human thought processes by providing an interface between perception, long-term memory and action. Consequently, the neural activity supporting WM underlies a broad range of advanced cognitive functions (Jonides et al., 2008).

The Y-maze spontaneous alternation task is one of the most widely used WM paradigms. Baeg et al. (2003) have found that firing rate changes of PFC neurons is tied to the reward and reward expectancy during a spatial delayed alternation task on a figure 8-shaped maze, implicating that WM is mediated by the PFC neuronal activities. Benchenane et al. (2010) have suggested that prefrontal cortex (PFC) is involved in rats learning new rules on a Y-maze both at the local field potential and spike levels. There is also evidence that PFC neuronal activity displays anticipatory changes prior to future goal-directed behaviors, suggesting that the PFC is responsible for holding neural representations for WM (Jo et al., 2013).

During a cognitive process, neuronal discharges are not independent but serially correlated with each other (Constantinidis and Goldman-Rakic, 2002). Therefore, a brain can be functionally defined as a network. Studies of functional network organization have become one of the most popular topics in the field of neuroscience (Palva et al., 2010; Sakaki et al., 2013). Several research has shown that cognitive performance was mediated by interactions among distributed, functionally specialized brain networks (White et al., 2011; Sala-Llonch et al., 2012). Conversely, specific network disruptions can cause a range of neurological disorders (Seeley et al., 2009). A powerful technique called Granger causality analysis could be used to extract such connections. Brain connectivity in humans or animals during cognitions has been analyzed using EEG (Anderson et al., 2010), fMRI (Schlösser et al., 2006), MEG (Bressler and Seth, 2011) and LFPs (Bressler et al., 2007), providing satisfactory explanations for neural mechanisms. These studies have opened new thought that the functional connectivity in mPFC networks may play an essential role in the WM. With the rapid development of chronic multi-electrode implantation, spikes can be recorded extracellularly. It is well-known that the spikes have better spatial and temporal resolutions than other signals. Therefore, these advances in the electrode implantation and brain network analyses have allowed us to map brain functional connectivity patterns *in vivo*.

A previous study has demonstrated that selective interaction among neurons is an essential component in neural information processing (Benchenane et al., 2010). Based on Hebb's theories, only a small fraction of neurons (“cell assembly”) participate in a task, selecting and preserving most valuable information to conduct future behaviors. Previous studies have demonstrated that sparse-coding is a convincing hypothesis to explain the way to process neural information (Sakurai, 1999; Schweighofer et al., 2001). Several studies have also reported that brain networks are sparse (Sporns et al., 2005; Hwang et al., 2013). However, most studies have only focused on functional connectivity among all recorded neurons (Lin et al., 2009; Nedungadi et al., 2009; Kim et al., 2011). It is cautioned that spurious effects may be introduced by neurons not in assemblies. From a network perspective, it is necessary to separate present vs. absent connections. Many investigators have attempted to extract the true network features by several mathematics methods. Recent work shows that cell assemblies can be extracted, which is used to represent the intrinsic pattern of neuronal activity (Peyrache et al., 2009; Benchenane et al., 2010). Up to now, non-negative matrix factorization (NMF), principal component analysis (PCA) and independent component analysis (ICA) have been already used to extract the latent structure of fMRI data (Lohmann et al., 2007; Shen and Meyer, 2008; Demirci et al., 2009; Zhou et al., 2009). In our study, NMF is employed to learn the parts-based components of realistic objects with non-negative constraints (Lee and Seung, 1999). The whole perception in brain relies on its parts-based representations (Logothetis and Sheinberg, 1996; Lee and Seung, 1999). And neuronal firing rates are never negative. Based on the theories, NMF has found its way into the neuroscience community (Lohmann et al., 2007; Padilla et al., 2010; Liu et al., 2013). It can be seen that the notion of combining parts to form a whole is intuitive and easy-to- interpreted. Here, NMF was used to remove dimensions that did not contribute to the raw firing rate pattern of mPFC neurons. The necessary information was correctly retained by NMF components. Therefore, the method could not only find intrinsic feature of neural activity, but also reduce computational cost.

In summary, a deeper understanding of the mPFC network changes may have profound implications for revealing the neural mechanism underlying WM. In this present work, we examined connectivity dynamics in mPFC spike networks in both NMF and original spaces, in order to investigate: (1) the association between the network changes and WM behavioral choices; (2) whether the network feature can be better represented in NMF space.

## Experimental procedures

### Subjects

Five male Sprague-Dawley rats weighting 300–350 g participated in this study. They were housed in temperature-controlled and clear plastic cages (25°C) under a 12:12-h light-dark schedule. All Experiments were conducted during the light phase. The experimental protocol was approved by the Committee on Experimental Animal of Tianjin Medical University.

### Apparatus

**Figure 1:**
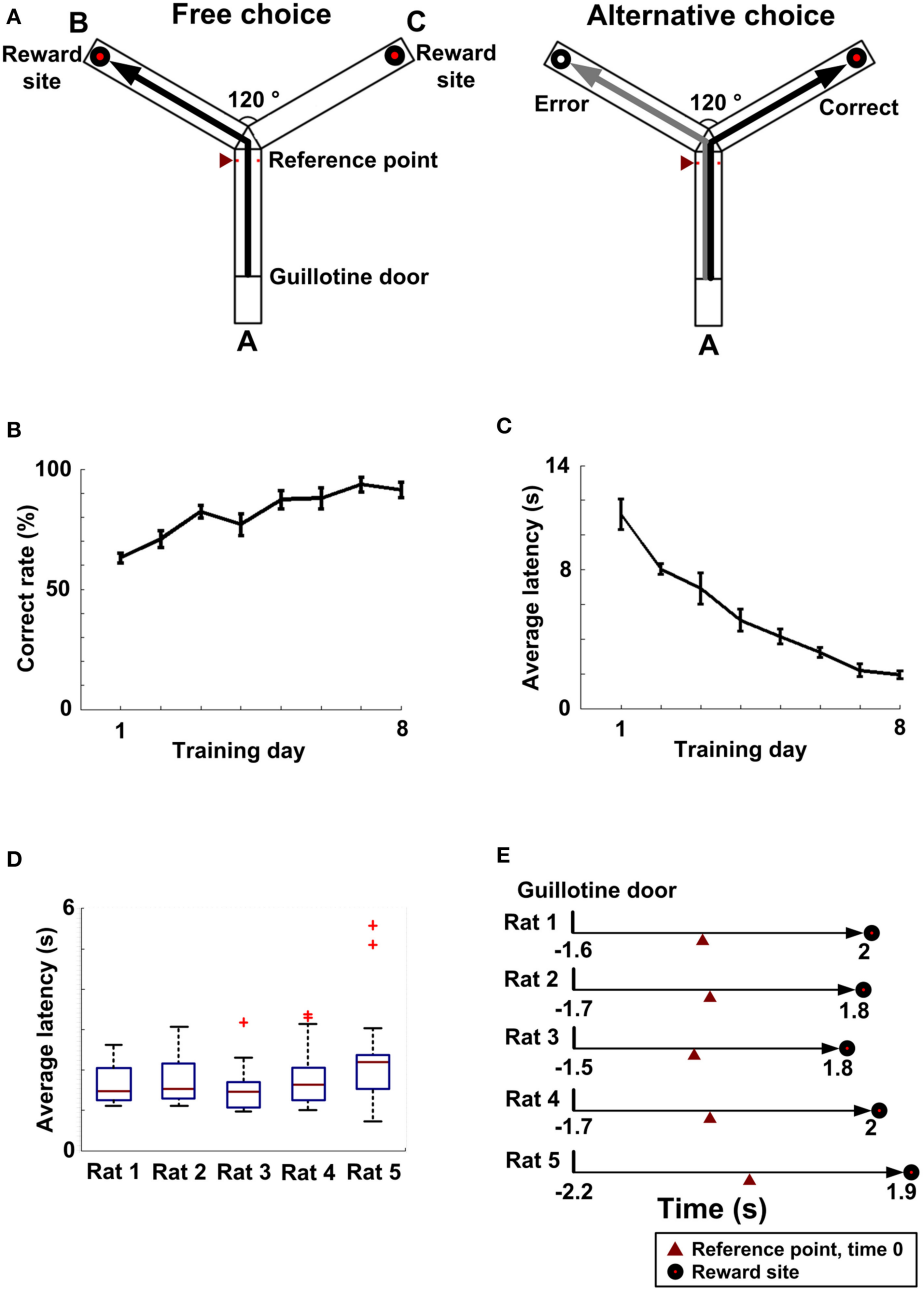
**Working memory behavior**. **(A)** A working memory task on a Y-maze (overhead view). An entire trial contained a free choice run (left) and an alternative choice run (right). Left: Arm A is a start arm. Reward sites are located at the ends of two goal arm B and C. RT was recorded by an infrared detector (red triangle). Right: A black line represents a correct choice and a gray line represents an error choice. **(B)** As training processed, the average rate of correct choice gradually increased (*n* = 5 rats). **(C)** As training continued, the average latency (*n* = 5 rats) gradually decreased. **(D)** The latency distribution (from the guillotine door to the choice point, *n*_Rat1_ = 24 trials, *n*_Rat2_ = 15 trials, *n*_Rat3_ = 21 trials, *n*_Rat4_ = 32 trials, *n*_Rat5_ = 22 trials) for each rat. **(E)** Average latency (from the guillotine door to the reward site, *n*_Rat1_ = 24 trials, *n*_Rat2_ = 15 trials, *n*_Rat 3_ = 21 trials, *n*_Rat4_ = 32 trials, *n*_Rat 5_ = 22 trials) over entire trials for each rat.

The apparatus was a Y-maze (Figure 1A), consisting of a start arm A and two goal arms B and C. Three arms were identical in size (80 cm long, 16 cm wide and 21 cm high) and other physical features. They were linked together in a symmetrical Y shape (120° of angular deviation from each other). A starting platform on the arm A was separated by a guillotine door. Two food wells were located at the end of each goal arm, respectively. Meanwhile, there was an infrared detector at the fork of the Y-maze to record a reference time (RT). The RT referred to the time for choice behavior.

### Behavioral experiments

The behavioral task was a spatial alternation Y-maze paradigm. First, all the rats were provided water *ad libitum* and food restriction for 2 days, maintaining at 85% of free-feeding body weight. Next, the rats explored the Y-maze and consumed reward food over two consecutive days (30 min per day). During habituation, they were forced to perform at least 15 entries to reward arms in 30 min. After that, the rats were trained to learn an alternative choice rule on the Y-maze. They were given one training session of 10 entire trials per day. An entire trial consisted of a free choice run and an alternative choice run. A latency for each trial referred to a period when a rat started from start point to reward site. During the free run, entering either goal arms was all rewarded. After ate, the rats ran back to the start location spontaneously and a guillotine door was closed. After 5 s delay, the door was lifted and the rats started to perform the choice run. They must choose a goal arm not previously entered to get reward. An error was defined by re-entering the arm visited in the last free run. On the way home, the arm that had not been visited was blocked by a guillotine door in order to prevent the rats quickly turning around and running into this arm. When the percent correct reached at least 80% over consecutive 2 days, the rats were considered as acquiring the rule used for the Y-maze task. If the rat has not learned the rule on day 8, it would be knocked out.

### Electrode implantation

After reaching the criterion of 80%, the rats were deeply anesthetized with sodium pentobarbital (40 mg/kg) supplemented with atropine (0.2 mg/kg) injected abdominally. Then a microelectrode array (2 × 0.3 mm2 in area, made in house) was chronically implanted in the medial PFC (mPFC, [AP: 2.5–4.5 mm; ML: 0.2–1 mm; DV: 2.5–3.5 mm]). This array consisted of 16 wires (2 × 8, 33 micron diameter nickel-cadmium, spaced 200 micron, <1 MΩ). During surgical procedures, animal's body temperature was monitored and maintained at 35°C~37°C. After the surgery, the rats were allowed to recover for at least 2 weeks. Food and water were available *ad libitum*.

### Electrophysiological recording and data preprocessing

Following 2 weeks recovery, the rats were again conducted into performed the Y-maze task. At the same time, neuronal activity was recorded. The RT for each trial was detected by an infrared sensor mounted on the Y-maze. The latencies for all trials were also recorded. To be clear, the trials mentioned in following paper referred to the alternative choice trials which could be used to measure the WM performance. A total 114 trials were analyzed (*n*_correct in total_ = 96, *n*_error in total_ = 18. Rat 1: *n*_correct_ = 20, *n*_error_ = 4; Rat 2: *n*_correct_ = 13, *n*_error_ = 2; Rat 3: *n*_correct_ = 18, *n*_error_ = 3; Rat 4: *n*_correct_ = 27, *n*_error_ = 5; Rat 5: *n*_correct_ = 18, *n*_error_ = 4) in the present paper. Besides, the baseline activity was taken during the inter-trial intervals. We compared the certain duration of mPFC neuronal activity (duration: 0.25 s pre—measure peak and 0.25 s post—measure peak) in correct trials with the baseline activity.

During the recording courses, the rats were connected to the Cerebus Acquisition system (Cyberkinetics, Foxborough, MA) via a rotating and lightweight cable. They were allowed to move freely on the Y-maze. Wide-band neural signals through the multi-electrode array were fed into a head stage amplifier with 5000 × gain and band-pass filtered between 250 and 7.5 kHz. The obtained signals whose amplitudes exceeded pre-set voltage thresholds were recorded, digitized at 30 kHz and stored for off-line spike sorting. Pre-set voltage thresholds for spike detection were done on negative amplitudes for each electrode independently. The threshold level was set as –7.5 times the standard deviation of raw signals (Reyes-Puerta et al., 2014). Any waveforms that crossed the corresponding threshold were detected and stored with time stamps. Since neural activity per channel was actually synthesized by several neurons nearby an electrode tip, Off-Line Sorter software (Plexon) was applied to extract multiple spike trains associated with different neurons by a semiautomatic clustering selection method (Lewicki, 1998) followed by manual clustering.

### Histology

After electro-physiological recordings, all the rats were sacrificed for histological analyses. The animals anaesthetized with sodium pentobarbital (40 mg/kg) were perfused intracardiacally with saline followed by 10% formalin saline. Then the brains were removed from the calvaria and trimming. Serial, 150 μm thick, coronal sections were carried out. Tracks and lesion sites were observed under a light microscope.

### Construction of a continuous time series from a spike train

Due to the discrete nature, spikes were not suitable for the subsequent connectivity analysis. A spike train must be converted into a continuous instantaneous firing rate time series (Zhu et al., 2005). The new series could encode the same temporal firing pattern of neuron as original spikes.

Specifically, the mathematical procedure consisted of three steps (Zhu et al., 2005). First, the instantaneous firing rate could be described by the following function:

(1) $$r(t)=\{\begin{array}{cc} 1/t_{i}-t_{i\;-\;1} & \;t_{i\;-\;1}\leq t\leq t_{i}\\ 1/t_{i\;+\;1}-t_{i} & \;t_{i}\leq t\leq t_{i\;+\;1} \end{array}$$

where *t*_*i* − 1_, *t_i_* and *t*_*i* + 1_ were considered as three successive spike times. Next, a small time interval δ*T* was chosen to obtain the instantaneous integrated rate as follow:

(2) $$f^{i}(t)=\int_{t}^{t\;+\;\delta T}r(t)dt\;t_{i\;-\;1}\leq t\leq t_{i\;+\;1}$$

Note that δ*T* was much smaller than the mean interspike interval *T*. According to the recommendation (Zhu et al., 2005), δ was set to 0.25. Finally, a continuous instantaneous firing rate series was yielded by smoothing out above functions in time (by Lagrange interpolation methods). In this way, each trial was represented by a matrix which consisted of *m* neurons of *n* instantaneous firing rates.

### Extracted components in NMF space

*X* is an *m*-by-*n* input matrix. *m* is the number of neurons. *n* is the number of instantaneous firing rates. NMF was performed on the input matrix to decompose it into non-negative basis matrix (*W*) and non-negative encoding matrix (*H*), as follows:

(3) $$X_{m\;\times \;n}\approx W_{m\;\times \;r}H_{r\;\times \;n}\;X,W,H\geq 0$$

(4) $$e=\min{\left\|X-WH\right\|}_{FRO}$$

where FRO is known as Frobenius norm and *e* is residual error matrix (Paatero and Tapper, 1994; Lee and Seung, 1999). *W* is a representation of NMF space. *H* is a low-dimensional representation of the input data (Padilla et al., 2010).

In NMF analysis, the estimation of rank *r* for factorization is a critical matter. It is required that at least:

(5) $$r<\frac{mn}{m+n}$$

The NMF computation was iterated by varying the parameter *r* from 1 to *m* (the number of neurons). The number whose variance accounted for (*VAF*) was just greater than 90% was selected as the rank *r* (Torres-Oviedo et al., 2006). The *VAF* is expressed as follows:

(6) $$VAF=1-\frac{\sum_{i\;=\;1}^{m}\sum_{j\;=\;1}^{n}{\left(e_{i,j}\right)}^{2}}{\sum_{i\;=\;1}^{m}\sum_{j\;=\;1}^{n}{\left(X_{i,j}\right)}^{2}}$$

For each trial, there was a NMF space. The *VAF* and rank *r* for each one were calculated. The rank *r* varied between animals, since different numbers of neurons were recorded as well as neurons with different contributions. There were also different ranks constructed from the same animal in different trials, as different neurons were either active or silent in different trials, or patterns of activity changed. In order to perform statistical analysis on data, maximum rank *r* for each rat was determined as the number of NMF components which keeps the *VAF* for each trial more than 90%. That means the neural activity pattern was well accounted for by extracted NMF components. Thus, the rank *r* was consistent within an animal, across trials. According to the final rank, we recalculated the corresponding *VAF* for each trial.

### Network analysis

#### Network construction

Granger causal connectivity analysis (GCCA) is a powerful approach for assessing functional connectivity among neural signals, revealing how one time series might exert influence on another (Zhou et al., 2013). If the past of one series does not contribute to the future of another one, the Granger causality (GC) between them is 0. When applied to a brain, this method has a potential to map the connectivity pattern of a brain network. A network is defined as a set of nodes linked by edges. In original space, nodes represent a set of neurons recorded in mPFC. In NMF space, nodes represent a group of NMF components. Edges represent the Granger causality values between their respective nodes. A 0.5-s moving window (moving step: 0.125 s) was applied to time series of every trials. Here we give a brief mathematical description about the Granger causality analysis (Seth, 2010). First, the multivariate time series can be described as follows:

(7) $$X={\left[x_{1}(t),x_{2}(t),\ldots ,x_{m}(t)\right]}^{T}$$

Next, a signal *X* is represented by a multivariate autoregressive model as follows:

(8) $$X(t)=\sum_{n\;=\;1}^{p}A(n)X(t-n)+E(t)$$

(9) $$E(t)={\left[\xi_{1}(t),\xi_{2}(t),\cdots ,\xi_{m}(t)\right]}^{T}$$

where *p* is the model order specified by the Bayesian information criterion, *A(n)* is the model coefficients, and *E(t)* contains the prediction errors for all time series, and ξ_*m*_(*t*) is the prediction error for *x_m_*. The *E*(*t*) covariance matrix of the unrestricted model is defined as follows:

(10) $$\begin{array}{l} \sum =\left[\begin{array}{cccc} var(\xi_{1U}) & cov(\xi_{1U}\xi_{2U}) & \cdots & cov(\xi_{1U}\xi_{mU})\\ cov(\xi_{2U}\xi_{1U}) & var(\xi_{2U}) & \cdots & cov(\xi_{2U}\xi_{mU})\\ \vdots & \vdots & \vdots & \vdots \\ cov(\xi_{mU}\xi_{1U}) & cov(\xi_{mU}\xi_{2U}) & \cdots & var(\xi_{mU}) \end{array}\right]\\ \;=\left[\begin{array}{cccc} \Sigma_{11} & \Sigma_{12} & \cdots & \Sigma_{1m}\\ \Sigma_{21} & \Sigma_{22} & \cdots & \Sigma_{2m}\\ \vdots & \vdots & \vdots & \vdots \\ \Sigma_{m1} & \Sigma_{m2} & \cdots & \Sigma_{mm} \end{array}\right] \end{array}$$

In a restricted model omitting variable *x_j_*, *E*(*t*) is represented as follows:

(11) $$E_{R}(t)={\left[\xi_{1}(t),\ldots \xi_{j\;-\;1}(t),\xi_{j\;+\;1}(t),\cdots ,\xi_{m}(t)\right]}^{T}$$

Thus, the *E_R_*(*t*) covariance matrix of the restricted model omitting *x_j_* is:

(12) $$\begin{array}{l} \rho =\left[\begin{array}{cccc} var(\xi_{1R}) & cov(\xi_{1R}\xi_{2R}) & \cdots & cov(\xi_{1R}\xi_{mR})\\ \vdots & \vdots & \vdots & \vdots \\ cov(\xi_{(j\;-\;1)R}\xi_{1R}) & cov(\xi_{(j\;-\;1)R}\xi_{2R}) & \cdots & cov(\xi_{(j\;-\;1)R}\xi_{mR})\\ cov(\xi_{(j\;+\;1)R}\xi_{1R}) & cov(\xi_{(j\;+\;1)R}\xi_{2R}) & \cdots & cov(\xi_{(j\;+\;1)R}\xi_{mR})\\ \vdots & \vdots & \vdots & \vdots \\ cov(\xi_{mR}\xi_{1R}) & cov(\xi_{mR}\xi_{2R}) & \cdots & var(\xi_{mR}) \end{array}\right]\\ \;=\left[\begin{array}{cccc} \rho_{11} & \rho_{12} & \cdots & \rho_{1m}\\ \vdots & \vdots & \vdots & \vdots \\ \rho_{(j\;-\;1)1} & \rho_{(j\;-\;1)2} & \cdots & \rho_{(j\;-\;1)m}\\ \rho_{(j\;+\;1)1} & \rho_{(j\;+\;1)2} & \cdots & \rho_{(j\;+\;1)m}\\ \vdots & \vdots & \vdots & \vdots \\ \rho_{m1} & \rho_{m2} & \cdots & \rho_{mm} \end{array}\right] \end{array}$$

The Granger causality from variable *x_j_* to variable *x_i_*, conditioned on all other variables is given by:

(13) $$\begin{array}{cc} F_{ij}=F_{x_{j}\to x_{i}}=\ln\frac{\rho_{ii}}{\Sigma_{ii}} & \left(i\neq j\right) \end{array}$$

Having constructed a Granger causality matrix, it is important to assess the statistical significance. Only significant connections were retained by *F-*tests (Seth, 2010; Frye et al., 2012). The tests were corrected for multiple comparisons by means of the false discovery rate (Benjamini and Hochberg, 1995). Connections which were not statistically significant were set to zeros.

#### Network properties

In the present study, we focus on the global properties in mPFC networks during the WM task. Both causal density (Cd) and global efficiency (E) can provide overall descriptions of a network.

Cd is a measure of causal interactions involving dynamical integration (neurons coordinate with each other) and differentiation (neurons contribute in different ways) among network nodes. Given a causal network identified by GCCA, Cd is defined as (Seth et al., 2006):

(14) $$Cd=Num_{GC}/N(N-1)$$

where *Num_GC_* is the total number of significant connections, *N* is the number of nodes. The values of *Cd* are bounded between 0 and 1. A neural network only containing independent neurons would have low *Cd*. By contrast, a network with high *Cd* indicates that nodes globally coordinate with each other and are dynamically distinct (Seth, 2010) to accomplish a task at the same time.

E is a measure of the capacity for nodes to propagate information in parallel across a network (Hwang et al., 2013). In general, the integration feature of a network is based on paths, which represent potential routes of information flow between pairs of nodes (Rubinov and Sporns, 2010). The path length is the sum of edge lengths along this path. Every edge length is determined by calculating the inverse of connection strength. The shortest path length between node *i* and *j* is the path with minimum total length (Yan et al., 2011). *E* could be defined mathematically as Bernhardt et al. (2011):

(15) $$E_{glob}=\frac{1}{N(N-1)}\sum_{i\;\neq \;j}\frac{1}{L_{ij}}$$

where *L_ij_* is the shortest path length between node *i* and *j*, *N* is the number of nodes. Previous studies had demonstrated that *E* was more suitable for sparser networks (Achard and Bullmore, 2007; Estrada and Hatano, 2008; Rubinov and Sporns, 2010). As the mPFC network in our study is sparse, inversing path length simplifies the numerical issues in the computation of *E* in sparser networks. Moreover, *E* measures the capacity to transfer parallel information. Since the brain supports large amounts of parallel information processing, *E* is a superior measure of network integration. A network having a high *E* suggests the existence of strong associations between nodes. Information is communicated with a high level of efficiency throughout the network.

### Statistical analysis

In this study, all data was expressed as mean ± standard error (SEM). Statistical analysis was carried out using One- or Two-Way ANOVA for repeated measures of the network features. *T*-test was used to compare the measures in the WM and baseline states. The significance level was set at *P* < 0.05.

### NMF-GCCA algorithm

In order to be more clearly, the general procedure for researching the mPFC network changes in original and NMF spaces was summarized as follows:

1. Obtain spike trains in rat mPFC during a WM task and convert each discrete train into a continuous time series.
2. Project multiple continuous series into a low-dimensional space by NMF, yielding NMF components which can represent the intrinsic pattern of mPFC neurons in original space.
3. Construct mPFC networks in NMF and original spaces using GCCA.
4. Analyze the properties of mPFC networks by computing *Cd* and *E*.
5. Repeat above steps for all trials of 5 rats.
6. Comparison of mPFC network dynamics during correct trials in both NMF and original spaces.
7. Compare mPFC network features between the certain WM and baseline states.
8. Compare mPFC network properties in correct and error trials.

## Results

### Working memory behavior

As training processed, the average correct rate (*n* = 5 rats) improved from 63 ± 5 to 91 ± 8% over 8 days (Figure 1B). And the average latency (*n* = 5 rats), from the start point to the choice point, gradually fell from 11.17 ± 0.88 s to 1.94 ± 0.12 s (Figure 1C). Finally, 5 rats successfully learned to alternate between the two goal arms to obtain reward (Figure 1A). By this time, the latency distribution (Rat 1: *n* = 24 trials; Rat 2: *n* = 15 trials; Rat 3: *n* = 21 trials; Rat 4: *n* = 32 trials; Rat 5: *n* = 22 trials) for each rat is shown in Figure 1D. The average latencies over a full trial were 1.6 ± 0.09 s, 1.7 ± 0.15 s, 1.5 ± 0.12 s, 1.7 ± 0.11 s, and 2.2 ± 0.25 s, respectively (Figure 1C).

### Neuronal activity in mPFC during working memory tasks

**Figure 2:**
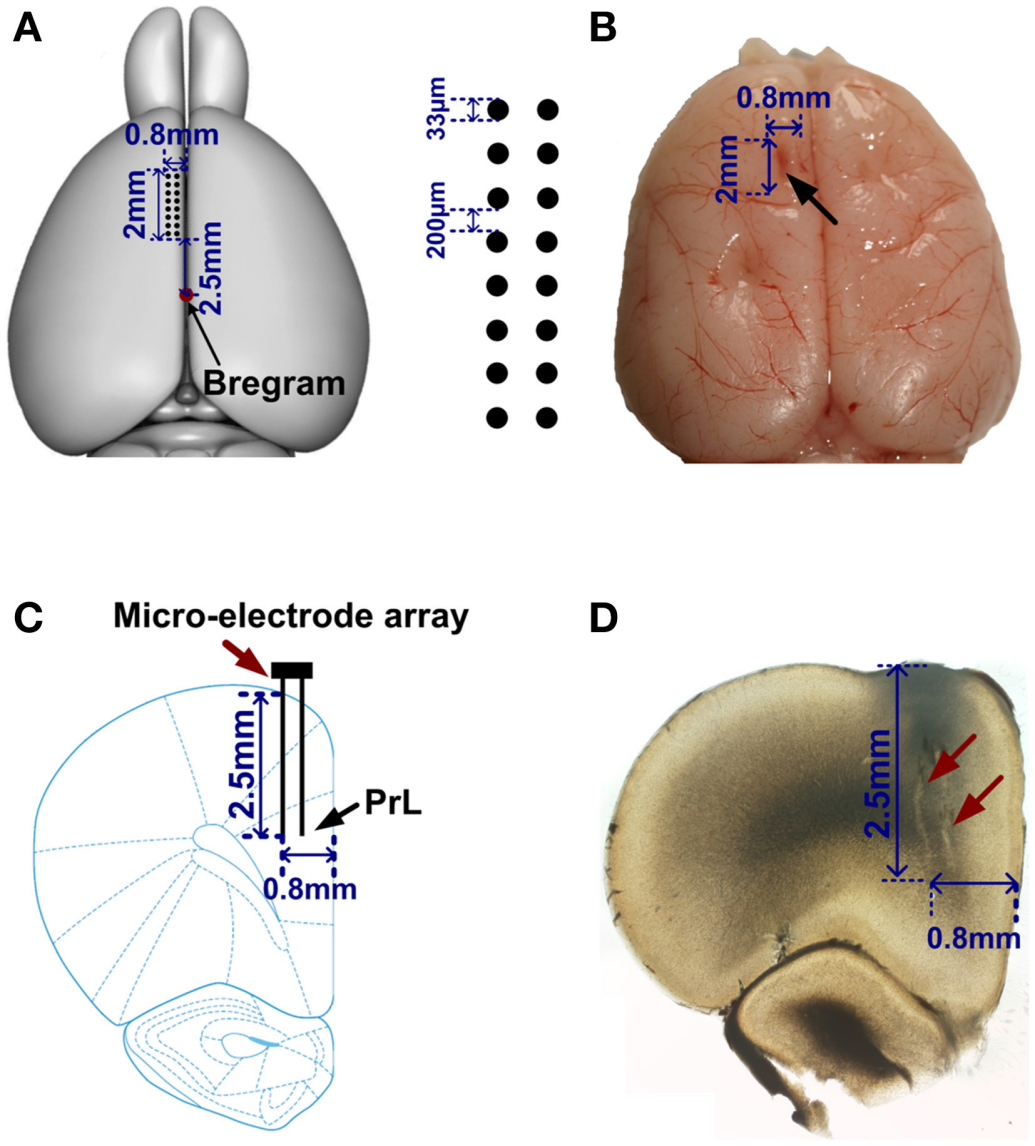
**Location of the electrode array in mPFC**. **(A)** Left, a rat brain model (overhead view). Red point indicates the Bregram. 2 × 8 black dots represent a micro-electrode array. **(B)** A representative photograph of a rat brain. The black arrow illustrates the extent of mPFC lesion after an electrode array was moved through this tissue. **(C)** A coronal section of a rat brain. An electrode array (red arrow) was implanted in PrL (black arrow). **(D)** Photomicrograph showing the tracks (red arrows) of recording electrodes.

**Figure 3:**
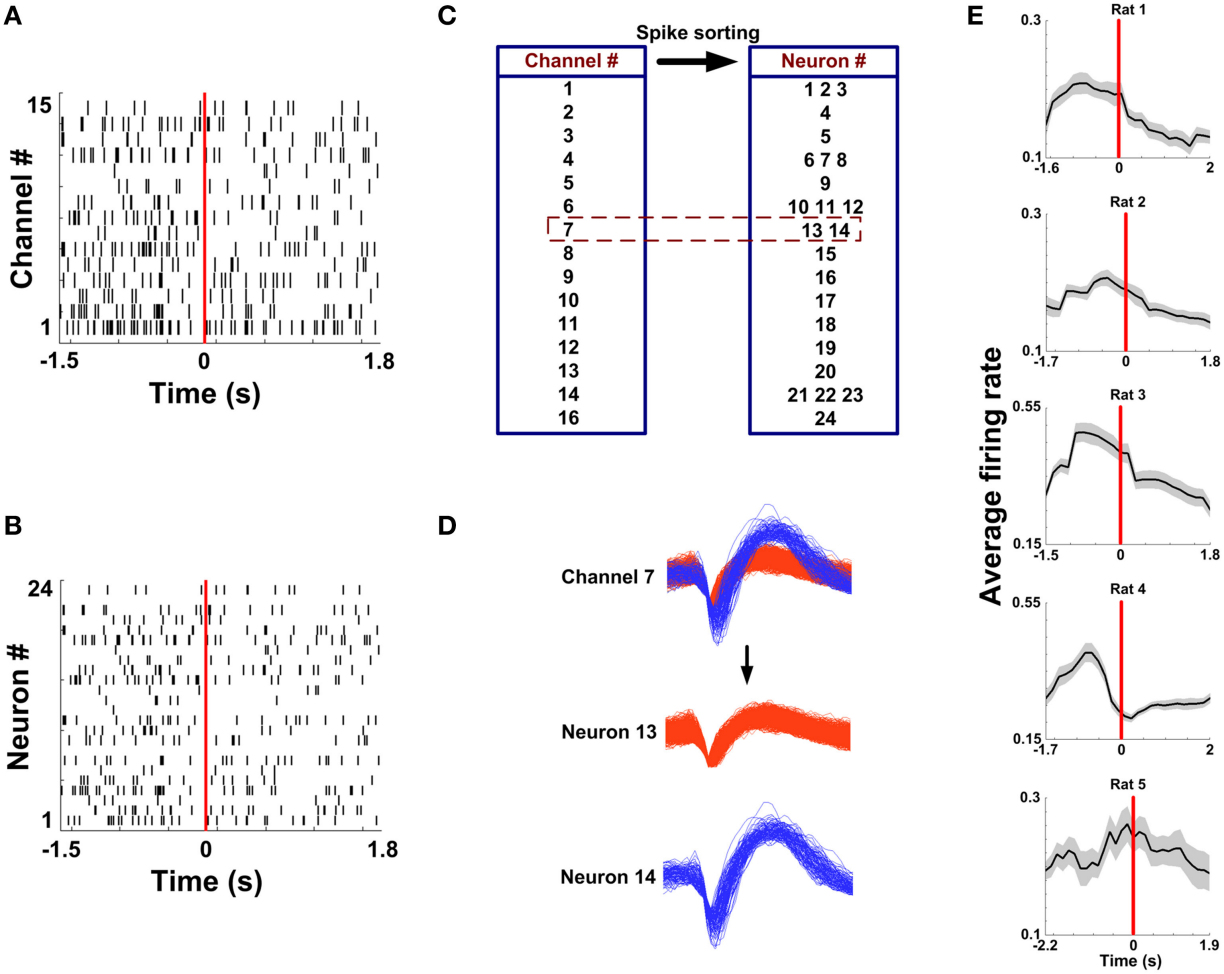
**Multi-channel spike sorting**. **(A)** Multi-unit spike trains from 15 channels during a correct trial. Time 0 (red vertical line) indicates the RT. **(B)** Single-unit spike trains sorted from the recordings in **(A)**. **(C)** A corresponding relationship between the electrode number and the neuron number. **(D)** Two neurons were isolated from the Channel 7. **(E)** Average firing rate as a function of time in correct trials for 5 rats (*n*_Rat1_ = 20 trials, *n*_Rat2_ = 13 trials, *n*_Rat3_ = 18 trials, *n*_Rat 4_ = 27 trials, *n*_Rat5_ = 18 trials).

Spikes in mPFC were recorded while rats performed WM tasks. Under a light microscope, the electrode tracks and lesion sites were unequivocally identified for 5 rats. The region of an implanted electrode array is provided in Figures 2A–D. Figure 3A displays representative spike activity in a correct trial. 15-channel spike trains were sorted, obtaining 24 single-unit spike trains (Figure 3B). Based on sorting results, the relationship between channel numbers and neuron numbers is shown in Figure 3C. For example, two neurons (neuron 13 and 14) were isolated from the Channel 7 (Figure 3D). Under this method, in total of 117 neurons (Rat 1: *n* = 17; Rat 2: *n* = 24; Rat 3: *n* = 32; Rat 4: *n* = 23; Rat 5: *n* = 21) were collected for analysis. The firing rates of correct trials were averaged using a series of sliding windows (width: 0.5 s, moving step: 0.125 s; Rat 1: *n* = 20 trials; Rat 2: *n* = 13 trials; Rat 3: *n* = 18 trials; Rat 4: *n* = 27 trials; Rat 5: *n* = 18 trials). One-Way ANOVA shows main effect of time on average firing rates (Rat 1—Rat 5, *p* < 0.05), indicating that the values significantly peaked before rats arriving at the maze choice point.

### Embedding dataset in NMF space

**Figure 4:**
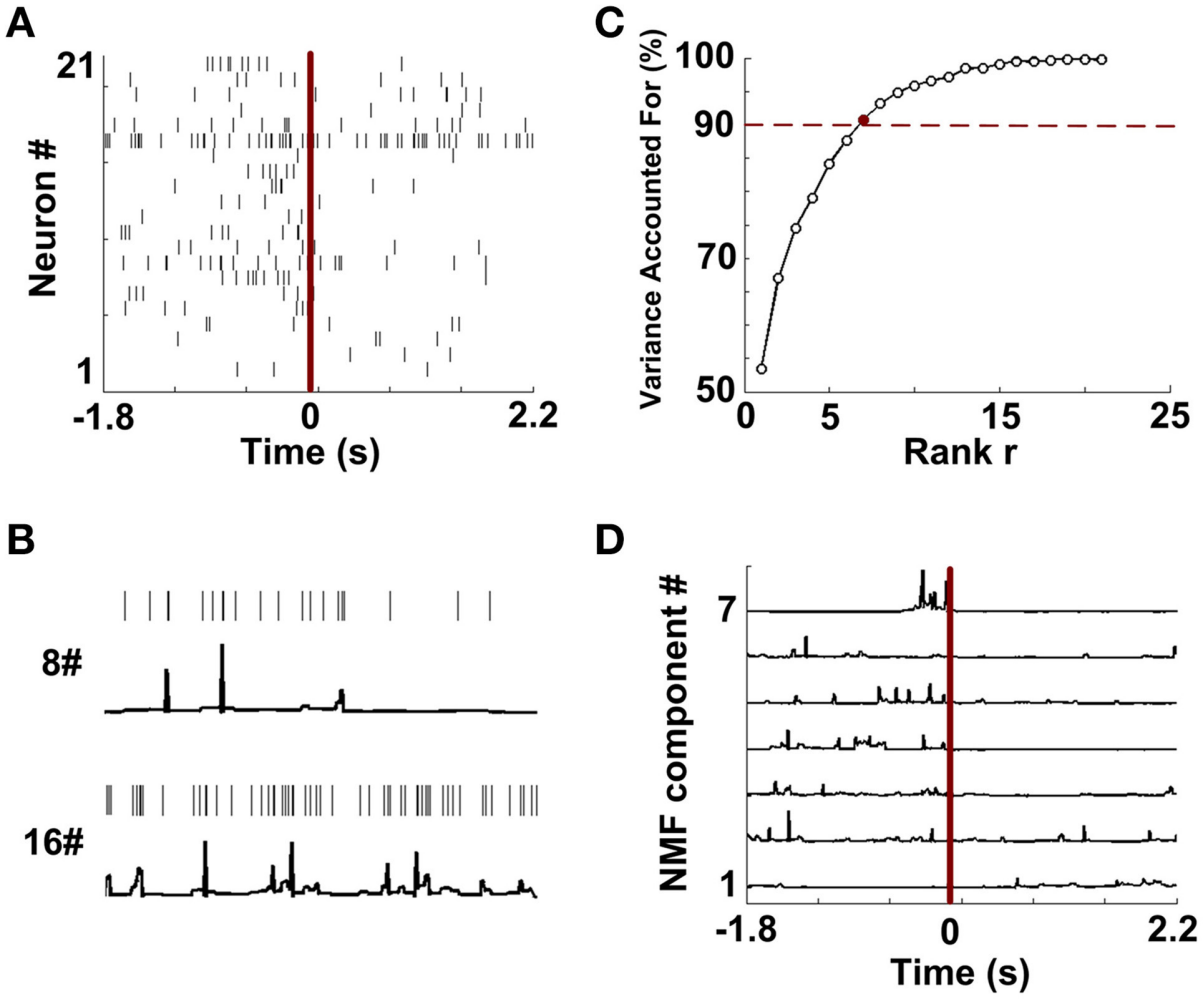
**Data preprocessing and NMF component extraction. (A)** The rastergram shows single-unit spike trains during a trial. **(B)** Two examples of constructions of continuous time series from discrete spike trains (Neuron 8 and 16 from **A**). **(C)** The percentages of Variance Accounted For with various number of rank r. Red dashed line means the threshold for *VAF* (90%). 7 (red point) is the optimized number of rank r in this trial. **(D)** NMF components extracted from the continuous series.

Figure 4A shows single-unit spikes during a WM trial. The spike trains were then converted into continuous-time series via the instantaneous firing rate method. Two representative signals (Neuron 8 and 16) are shown in Figure 4B. The results suggest that continuous series can describe the temporal firing patterns of neurons. Then the resulting time series were embedded in NMF space. Figure 4C depicts that *VAF* varied with rank *r*. By using the criterion previously described, the data shown in Figure 4A has a rank of 7. According to the selected rank, 7 NMF components were identified from this trial (Figure 4D).

In order to keep a same rank across trials in a rat, the maximum rank *r* was chosen to be a final rank *r* (Figure S1). They were 5, 7, 10, 8, and 7 for Rat #1, #2, #3, #4, and #5. The corresponding mean *VAF* was 95.1 ± 0.84% (Rat 1, *n* = 20 trials), 96.3 ± 0.92% (Rat 2, *n* = 13 trials), 91.8 ± 0.26% (Rat 3, *n* = 18 trials), 95.7 ± 0.47% (Rat 4, *n* = 27 trials) and 92.9 ± 0.48% (Rat 5, *n* = 18 trials), respectively. The results show that based on the maximum rank, neural activity was well accounted for by extracted NMF components.

### Changes of mPFC networks during correct trials in original and NMF spaces

**Figure 5:**
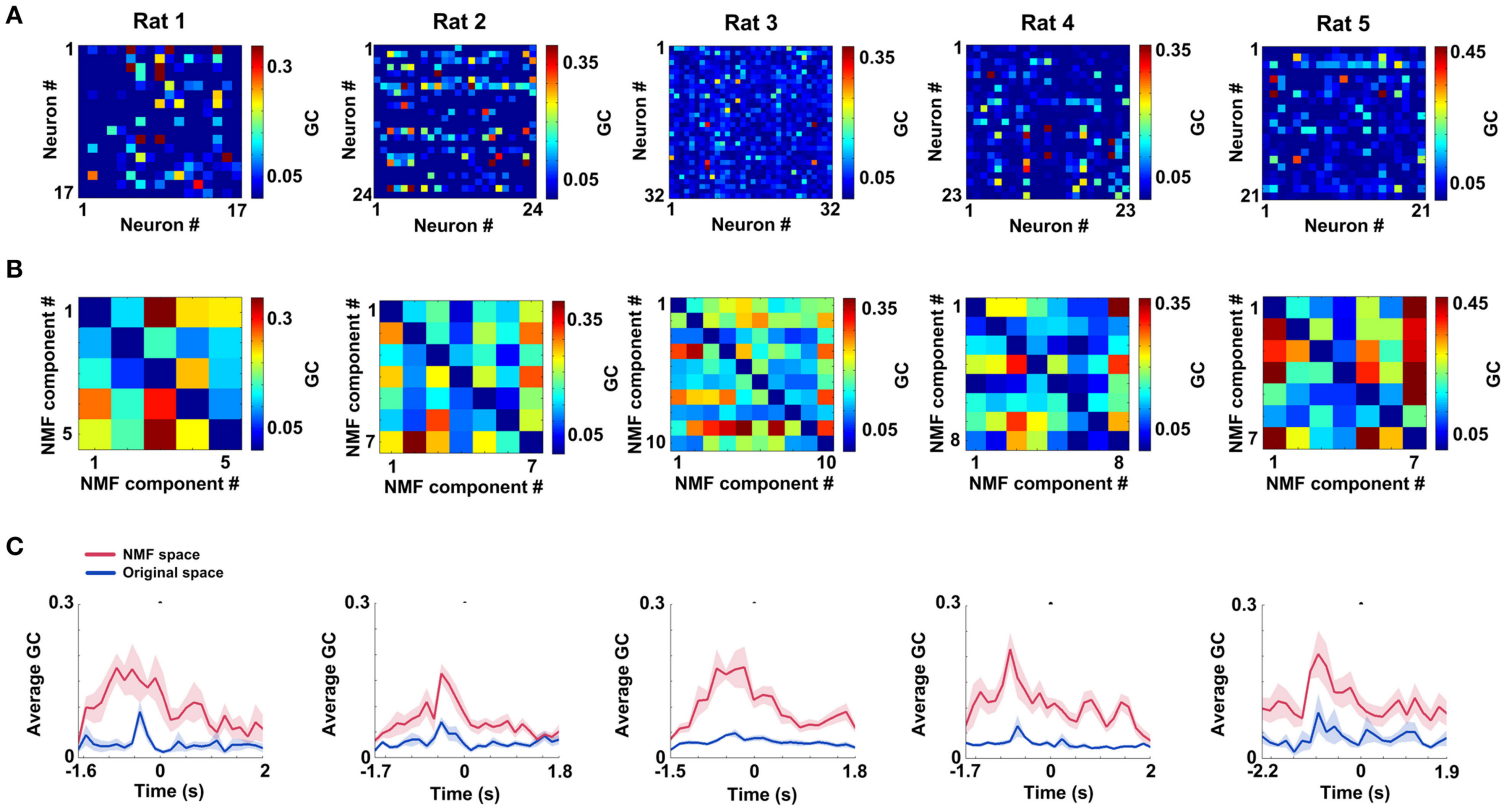
**mPFC spike networks during WM in original and NMF spaces. (A)** Mean connectivity matrices during WM for 5 rats in original space (*n*_Rat1_ = 20 trials, *n*_Rat 2_ = 13 trials, *n*_Rat 3_ = 18 trials, *n*_Rat 4_ = 27 trials, *n*_Rat5_ = 18 trials). Each node represents a neuron. Colored grid represents the Granger causality between each pair of neurons. The hot colors show strong connections and the cold ones show weak connections. **(B)** Mean connection matrices in NMF space (*n*_Rat 1_ = 20 trials, *n*_Rat 2_ = 13 trials, *n*_Rat 3_ = 18 trials, *n*_Rat 4_ = 27 trials, *n*_Rat 5_ = 18 trials). Each node represents a NMF component. Colored grid represents the Granger causality between each pair of NMF components. The grids are coded according to the connection weight. **(C)** Average Granger causality weights as a function of time in correct trials for 5 rats (red: NMF space, blue: original space; *n*_Rat 1_ = 20 trials, *n*_Rat 2_ = 13 trials, *n*_Rat 3_ = 18 trials, *n*_Rat 4_ = 27 trials, *n*_Rat 5_ = 18 trials).

mPFC networks in both original and NMF spaces were constructed by GCCA method. The mean connectivity matrices (duration: 0.25 s pre—measure peak and 0.25 s post—measure peak) during correct trials in two spaces are shown in Figure 5A (original space) and Figure 5B (NMF space) for 5 rats (Rat 1: *n* = 20 trials; Rat 2: *n* = 13 trials; Rat 3: *n* = 18 trials; Rat 4: *n* = 27 trials; Rat 5: *n* = 18 trials). From visual inspection, the matrices in NMF space appear to exhibit strong connections. The subsequent statistics for results suggest that mean connection values during the certain WM durations in NMF space (Rat 1, 0.15 ± 0.015; Rat 2, 0.12 ± 0.010; Rat 3, 0.15 ± 0.01; Rat 4, 0.14 ± 0.01; Rat 5, 0.15 ± 0.013) are significantly greater than those in original space (*t*-test, Rat 1, 0.04 ± 0.005, *p* < 0.01; Rat 2, 0.04 ± 0.005, *p* < 0.01; Rat 3, 0.04 ± 0.002, *p* < 0.01; Rat 4, 0.04 ± 0.003, *p* < 0.01; Rat 5, 0.06 ± 0.009, *p* < 0.01). Additionally, the mean connection weights as a function of time in correct trials are shown in Figure 5C. It can be found that the measures peak before correct behavioral choices. And the changes of mean connection weights in NMF and original spaces appear to track one another. However, compared with the values in original space, the NMF analysis did increase the average connection weights (Two-Way ANOVA, factors: NMF / original space (S) and time on maze (T); Rat 1, *n* = 20 trials, main effect: S: *p* < 0.05, T: *p* < 0.05, interaction *p* < 0.05; Rat 2, *n* = 13 trials, main effect: S: *p* < 0.01, T: *p* < 0.05, interaction *p* < 0.05; Rat 3, *n* = 18 trials, main effect: S: *p* < 0.01, T: *p* < 0.01, interaction *p* < 0.01; Rat 4, *n* = 27 trials, main effect: S: *p* < 0.01, T: *p* < 0.01, interaction *p* < 0.01; Rat 5, *n* = 18 trials, main effect: S: *p* < 0.01, T: *p* < 0.01, interaction *p* < 0.05), especially near the peaks.

### Causal density dynamics of mPFC networks during correct trials in original and NMF spaces

**Figure 6:**
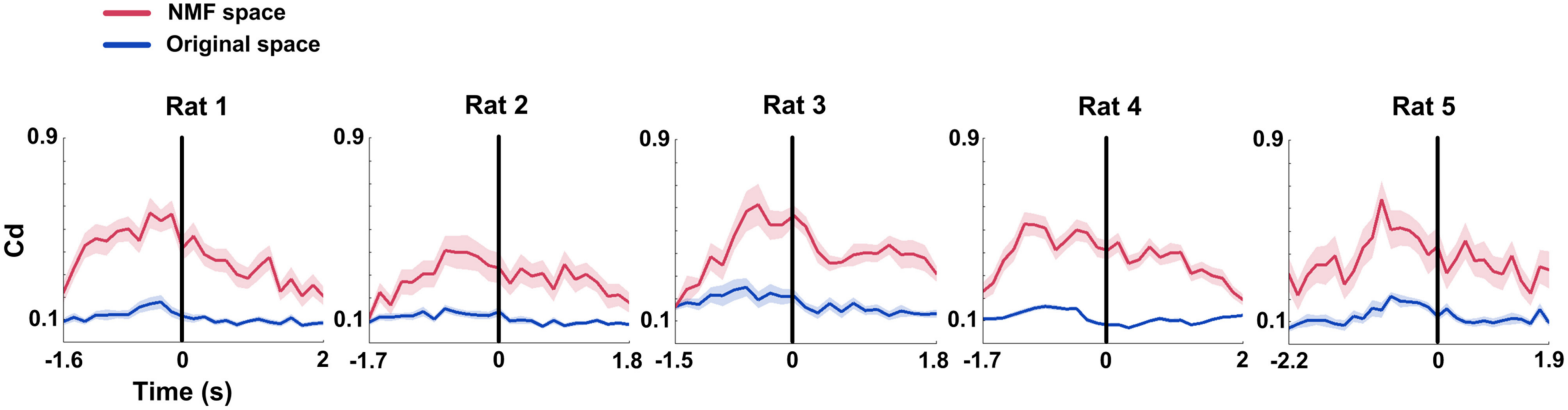
**Causal density (Cd) of mPFC networks during correct WM trials in original and NMF spaces**. Red lines: NMF space. Blue lines: original space (*n*_Rat 1_ = 20 trials, *n*_Rat 2_ = 13 trials, *n*_Rat 3_ = 18 trials, *n*_Rat 4_ = 27 trials, *n*_Rat 5_ = 18 trials). *Cd* values in the two spaces display a similar changing tendency across correct trials. They increased to peaks before the RT (time 0, black vertical line). Cd in NMF space was significantly higher than that in original space. *Abscissa*: time course that the rat ran from the start point to the reward site. Shaded area indicates the SEM.

Similar to the mean connection weights, Causal density (Cd) increased to peaks in the interval right before rats' correct behavioral choices. The distinct increases were observed in both original and NMF spaces for 5 rats (Figure 6, from left to right; Two-Way ANOVA, factors: NMF / original space (S) and time on maze (T); Rat 1, *n* = 20 trials, main effect: S: *p* < 0.01, T: *p* < 0.05, interaction *p* < 0.05; Rat 2, *n* = 13 trials, main effect: S: *p* < 0.01, T: *p* < 0.01, interaction *p* < 0.05; Rat 3, *n* = 18 trials, main effect: S: *p* < 0.01, T: *p* < 0.01, interaction *p* < 0.01; Rat 4, *n* = 27 trials, main effect: S: *p* < 0.01, T: *p* < 0.01, interaction *p* < 0.01; Rat 5, *n* = 18 trials, main effect: S: *p* < 0.01, T: *p* < 0.05, interaction *p* < 0.05). Additionally, Cd in NMF space was significantly higher than that in original space. The results indicated that the changes of Cd in correct trials were represented more obvious by NMF analysis.

### Global efficiency changes of mPFC networks during correct trials in original and NMF spaces

**Figure 7:**
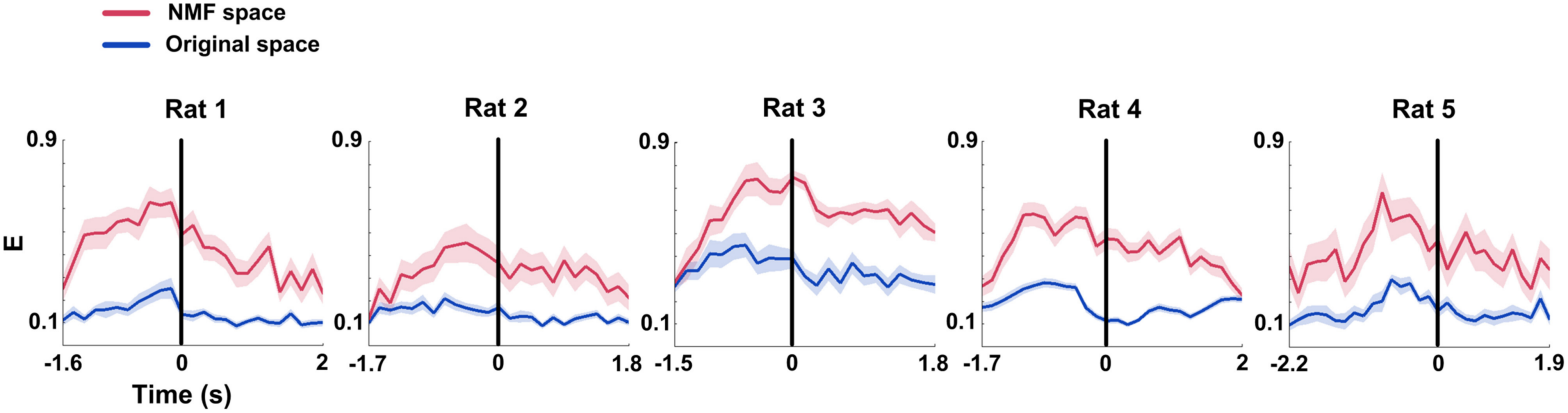
**Global efficiency (E) features of mPFC spike network during correct WM trials in original and NMF spaces**. Red lines: NMF space. Blue lines: original space (*n*_Rat 1_ = 20 trials, *n*_Rat 2_ = 13 trials, *n*_Rat 3_ = 18 trials, *n*_Rat 4_ = 27 trials, *n*_Rat 5_ = 18 trials). E increased to peaks prior to the maze choice point in both of the two spaces. The difference was that the mean E in NMF space stayed above the values in original space. *Abscissa*: time course that the rat ran from the start point to the reward site. Shaded area indicates the SEM.

Global efficiency (E) changes of mPFC networks were examined in both original and NMF spaces for each rat (Figure 7). During correct trials, we found the trends of increased E occurring before the choice point in two spaces (Figure 7, from left to right; Two-Way ANOVA, factors: NMF / original space (S) and time on maze (T); Rat 1, *n* = 20 trials, main effect: S: *p* < 0.01, T: *p* < 0.05, interaction *p* < 0.05; Rat 2, *n* = 13 trials, main effect: S: *p* < 0.01, T: *p* < 0.05, interaction *p* < 0.05; Rat 3, *n* = 18 trials, main effect: S: *p* < 0.01, T: *p* < 0.01, interaction *p* < 0.01; Rat 4, *n* = 27 trials, main effect: S: *p* < 0.01, T: *p* < 0.01, interaction *p* < 0.01; Rat 5, *n* = 18 trials, main effect: S: *p* < 0.01, T: *p* < 0.01, interaction *p* < 0.05). The statistical results indicate that there were profound modifications in E of mPFC networks during correct trials. And like the Cd, E in NMF space was significantly greater than that in original space, indicating that the WM-related mPFC network dynamics was highlighted in NMF space.

### mPFC network properties in certain WM and baseline states

**Figure 8:**
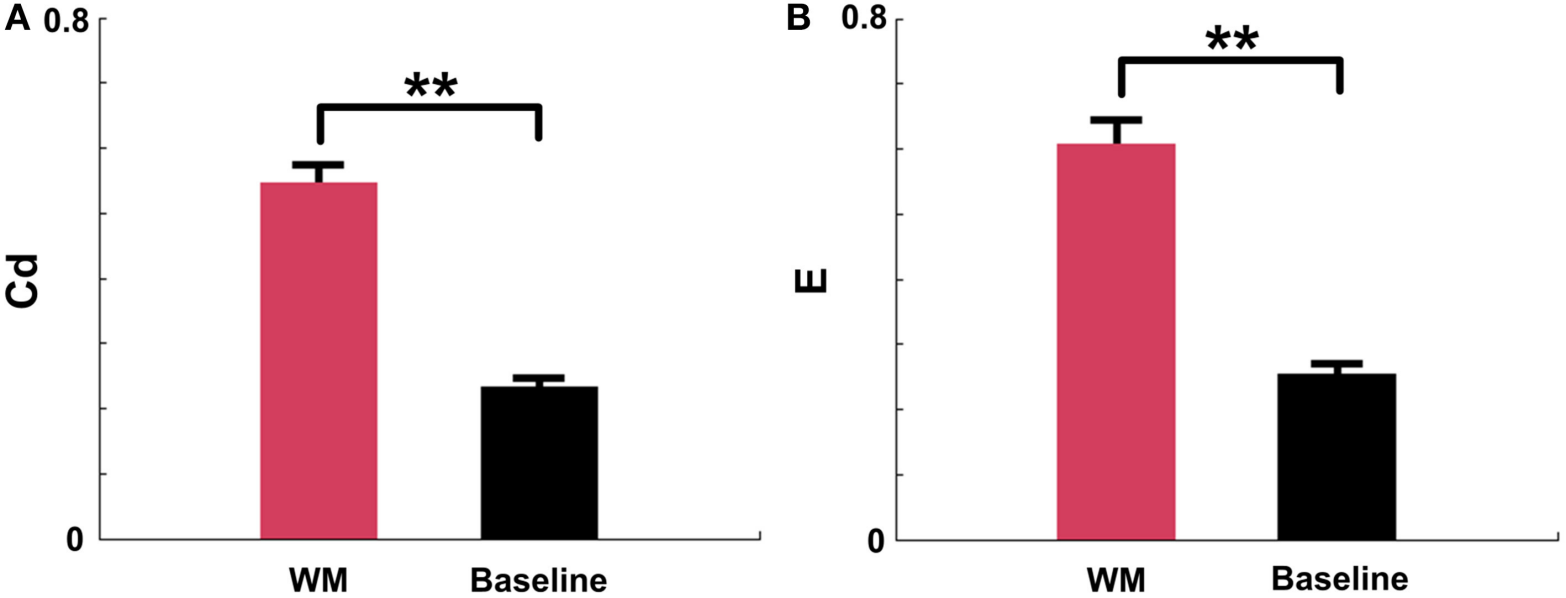
**Causal density (Cd) and global efficiency (E) of mPFC networks in certain WM and baseline states in NMF space**. Red: certain WM states. Black: baseline states. **(A)** Cd of certain WM states in correct trials (duration: 0.25 s pre-measure peak and 0.25 s post-measure peak, *n* = 96 trials, 5 rats) is significantly greater than that in baseline states (*n* = 91 trials, 5 rats, *t*-test, ^**^p < 0.01). **(B)** E of certain WM states in correct trials (duration: 0.25 s pre-measure peak and 0.25 s post-measure peak, *n* = 96 trials, 5 rats) is significantly higher than that in baseline states (*n* = 91 trials, 5 rats, *t*-test, ^**^p < 0.01).

The above results demonstrated that the Cd and E in mPFC networks significantly increased before the maze choice point during correct trials. In order to examine whether the increases were meaningful changes, we compared the measures in certain periods (duration: 0.25 s pre—measure peak and 0.25 s post—measure peak, *n* = 96 trials, 5 rats) in correct trials to measures in the baseline states (*n* = 91 trials, 5 rats). As shown in Figure 8, *t*-test reveals that there are significant differences between the certain WM and baseline groups (Figure 8A, Cd: *p* < 0.01; Figure 8B, E: *p* < 0.01).

### mPFC network properties in correct and error WM trials

**Figure 9:**
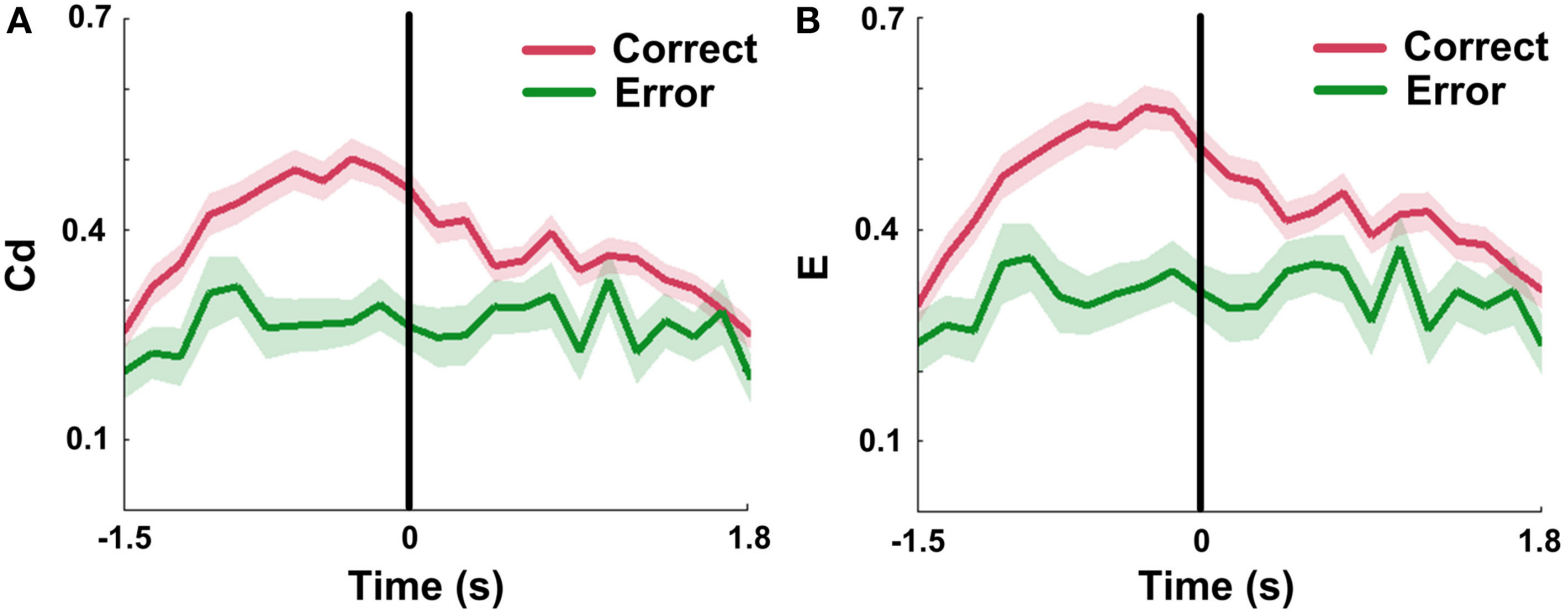
**mPFC network properties in correct and error trials in NMF space**. Red: correct trials. Green: error trials. **(A)** Mean Cd in correct (*n* = 96, 5 rats) vs. error (*n* = 18, 5 rats) trials. No significant increase was observed in error trials. *Abscissa*: the minimum average latency among 5 rats. Shaded area indicates the SEM. **(B)** Average E in correct (*n* = 96, 5 rats) vs. error (*n* = 18, 5 rats) trials. The values showed a slight concussion across the whole trials. *Abscissa* and shaded area: Same as in **(A)**.

Since Cd and E in mPFC networks changed dynamically during correct trials, we attempted to ascertain the network properties when rats made error choices. The results show that measures in error groups are significantly lower than those in correct groups (Figures 9A,B; *n*_Correct_ = 96 trials; *n*_Error_ = 18 trials, Two-Way ANOVA, factors: correct/error choice (C) and time on maze (T); Cd, main effects: C: *p* < 0.01, T: *p* < 0.01, interaction *p* > 0.05; E, main effects: C: *p* < 0.01, T: *p* < 0.01, interaction *p* > 0.05; 5 rats). And the Cd and E almost stayed constant with slight oscillation over entire time courses. One-Way ANOVA was then applied to determine whether there was a main effect of time on the measures in error trials. The results indicate that no distinct changes were found in error trials (*n*_Error_ = 18 trials, One-Way ANOVA: Cd: *p* > 0.05; E: *p* > 0.05; 5 rats).

## Discussion

This present study explored mPFC network changes during a WM task, employing multi-channel spikes. We embedded spike trains into a NMF space, followed by network analysis in this transformed space. The principle findings here are (1) Cd and E of mPFC networks changed dynamically while the rats correctly performed WM tasks; (2) before the rats arrived at the maze choice point, Cd and E had already augmented to peaks; (3) mPFC network properties were highlighted by NMF analysis. Furthermore, mPFC network measures of certain WM states in correct trials are significantly higher than measures in baseline states. And we did not observe increased network measures in error trials.

The prefrontal cortex is at the top of the cortical perception-action cycle. WM is supported by this high-order brain area. In the Y-maze task, rats must keep reward location in free choice run “in mind” for short periods, which is used to execute subsequent alternative choices. With current multi-unit recording techniques, we can simultaneously record spikes from many neurons and track their activity changes directly. Previous studies have demonstrated that neurons in mPFC maintain WM-related information through their synaptic connections (Hains et al., 2009; Paspalas et al., 2013). The connections enable mPFC networks to efficiently process WM-related information. By studying the functional properties of mPFC networks, we may gain insight into the true nature of mPFC that underlies WM. For recent years, neurophysiologists have studied functional interactions among neurons during special cognitions (Lin et al., 2009; Stevenson et al., 2009; Hirabayashi et al., 2010; Masud and Borisyuk, 2011). In our study, well trained rats made choices at the maze choice point, on the basis of a prediction about which goal arm was the most likely rewarded one. The results show that Cd and E of mPFC networks change dynamically in correct trials. When rats started from the start point, the measures were gradually rising over time. Their peaks all appeared before the choice point. Then the measures begun to fall after rats entered the goal arms. The statistical results suggested that Cd or E in the certain durations (0.25 s pre—measure peak and 0.25 s post—measure peak) of correct trials was significantly higher than the measures in baseline states, indicating that there were real changes in Cd and E. Additionally, the significant increase pattern was not found during runs toward the choice point in error trials. mPFC network dynamics was different between correct and error trials, suggesting an association between the network states and correct behavioral choices. It is inferred that increases in mPFC network measures might have an influence as rats assessed the possible results of choices, consistent with a previous WM study (Rossetti and Carboni, 2005).

Methods for accurately estimating functional connectivity may be important for many neuroscience issues. Brain neural activity is sparse which is the point of the feature space transformation. Low-dimensional representations chosen from high dimensional data can preserve or highlight some interested features in the data (Cunningham and Yu, 2014). NMF is one of the dimensionality reduction methods. Previous studies had demonstrated that NMF could extract the dominant features in fMRI (Lohmann et al., 2007), EMG (Chiovetto et al., 2013), SPECT (Padilla et al., 2010), ECG (Ghoraani et al., 2011) database and odor profiles (Castro et al., 2013). In our study, the network analysis in NMF space presented here approaches an efficient description of mPFC network properties. The results in Figure 5A show that mPFC networks in original space are naturally sparse, in that many Granger causality values are exactly equal to zero. It is possible that some dimensions are redundant, resulting in the latent neuronal activity being confined to a low-dimensional slice of original space. NMF was used to remove dimensions that did not contribute to the raw firing rate pattern of mPFC neurons. The necessary information was correctly retained by NMF components. In Figure 5B, the average connections of mPFC networks are significantly greater than those in Figure 5A, indicating that featured mPFC activity may densely occupy the NMF space. Therefore, the functional connectivity among NMF components may offer a more accurate description of mPFC network features. The dynamics of Cd (or E) in two spaces tracking one another means that NMF components preserve the necessary information in original data. Higher Cd (or E) means that the network properties in NMF space are more apparent. The results suggested that NMF analysis faithfully captured the patterns of mPFC networks and made them more obvious. Our approach enables us to study network dynamics from a novel perspective that are not observable in original space, opening a possibility of better identifying the network characteristics.

Numerous researches have shown that mPFC is linked with WM. In the present study, food-restricted rats were rewarded if they chose the goal arm not entered in the previous free run. In this process, mPFC recalled and integrated task-relevant information to guide behavior appropriately. Previous electrophysiological evidence has suggested that lesion or inactivation of mPFC often caused increased error responding and longer retention delays in rats performing delayed spatial alternation tasks (Wang and Cai, 2006; Yoon et al., 2008; Horst and Laubach, 2009). From a molecular and cellular perspective, Kim and colleagues also have revealed that mPFC is responsible for WM by using mice with mPFC-selective PLC-β1-knockdown (Kim et al., 2014). When carried out a series of behavioral tests, they found that the mice with impaired mPFC were incapable of acquiring the task. However, rats did not depend only on the WM to perform trials during the Y-maze task. The other process might be involved. For instance, rats should inhibit immature response to the previously rewarded arm to get reward in the alternative choice run. Therefore, whether the observed mPFC activity also presumably reflects and drives many aspects of behavior (such as performance monitoring and cognitive control) deserves discussion. Previous study has suggested that the activity of a significant percentage of the mPFC neurons did represent and maintain prior task-relevant information to guide appropriate WM task behavior (Jones and Wilson, 2005). Moreover, a well-controlled study has demonstrated that elevated delay-period activity in mouse mPFC appeared to play a more important role in memory retention than inhibitory control, motor selection, and decision-making (Liu et al., 2014). These studies, together with the results reported here, support that the recorded mPFC activity closely linked with WM during Y-maze tasks.

## Conclusions

In summary, we have studied changes of mPFC spike networks during WM by means of NMF analysis. The findings point to a possible mechanism wherein the significant increases in mPFC network measures might conduct rats' subsequent choice behaviors. The anticipatory changes can be predictors of rat's future WM performance. Further results indicated that by considering the sparse feature of neural activity, the NMF method can provide a better characterization of network dynamics with less computational cost.

### Conflict of interest statement

The authors declare that the research was conducted in the absence of any commercial or financial relationships that could be construed as a potential conflict of interest.
