# Supplementary figures and images for: Functional connectivity changes during a working memory task in rat via NMF analysis
Source: Front Behav Neurosci. 2015 Jan 30;9:2. doi: 10.3389/fnbeh.2015.00002 (PMC4311635; doi:10.3389/fnbeh.2015.00002)

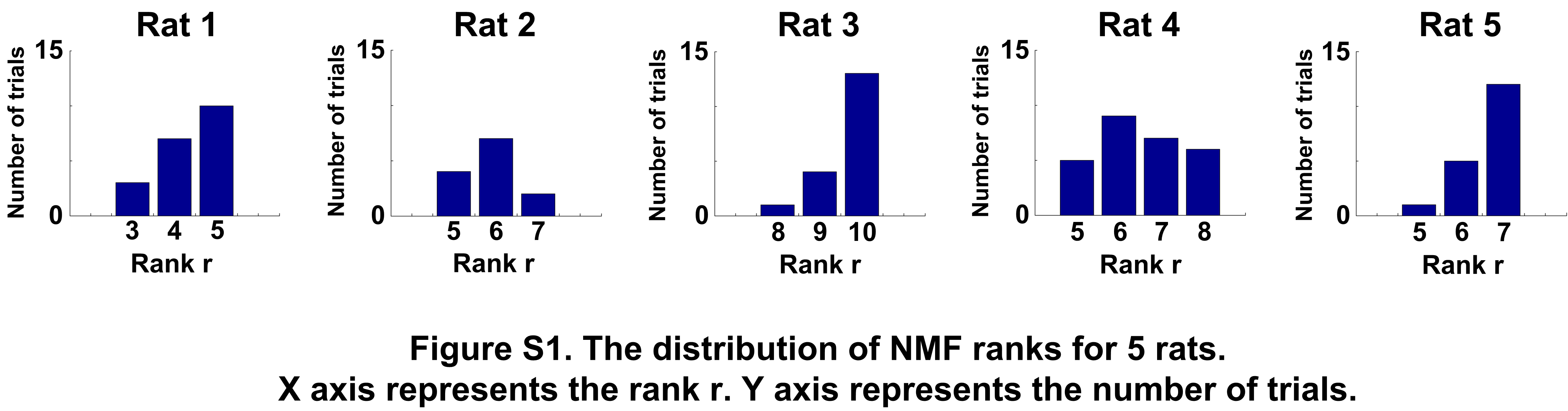

Supplement: Supplementary file 1 [file Image1.TIF]
